# Molecular and functional characterization of ferredoxin NADP(H) oxidoreductase from *Gracilaria chilensis* and its complex with ferredoxin

**DOI:** 10.1186/s40659-017-0144-5

**Published:** 2017-12-08

**Authors:** María Alejandra Vorphal, Carola Bruna, Traudy Wandersleben, Jorge Dagnino-Leone, Francisco Lobos-González, Elena Uribe, José Martínez-Oyanedel, Marta Bunster

**Affiliations:** 1 Laboratorio de Biofísica Molecular, Departamento de Bioquímica y Biología Molecular, Facultad de Ciencias Biológicas, Universidad de Concepción, Barrio Universitario S/N, Casilla 160_C, Concepción, Chile; 2 Laboratorio de Enzimología, Departamento de Bioquímica y Biología Molecular, Facultad de Ciencias Biológicas, Universidad de Concepción, Barrio Universitario S/N, Casilla 160_C, Concepción, Chile

**Keywords:** Ferredoxin NADP^+^ reductase, Ferredoxin, Sequence, Kinetic parameters, Structural features

## Abstract

**Backgroud:**

Ferredoxin NADP(H) oxidoreductases (EC 1.18.1.2) (FNR) are flavoenzymes present in photosynthetic organisms; they are relevant for the production of reduced donors to redox reactions, i.e. in photosynthesis, the reduction of NADP^+^ to NADPH using the electrons provided by Ferredoxin (Fd), a small FeS soluble protein acceptor of electrons from PSI in chloroplasts. In rhodophyta no information about this system has been reported, this work is a contribution to the molecular and functional characterization of FNR from* Gracilaria chilensis*, also providing a structural analysis of the complex FNR/Fd.

**Methods:**

The biochemical and kinetic characterization of FNR was performed from the enzyme purified from phycobilisomes enriched fractions. The sequence of the gene that codifies for the enzyme, was obtained using primers designed by comparison with sequences of* Synechocystis* and EST from* Gracilaria*. 5′RACE was used to confirm the absence of a CpcD domain in FNRPBS of* Gracilaria chilensis*. A three dimensional model for FNR and Fd, was built by comparative modeling and a model for the complex FNR: Fd by docking.

**Results:**

The kinetic analysis shows K_M_^NADPH^ of 12.5 M and a* k*_cat_ of 86 s^−1^, data consistent with the parameters determined for the enzyme purified from a soluble extract. The sequence for FNR was obtained and translated to a protein of 33646 Da. A FAD and a NADP+ binding domain were clearly identified by sequence analysis as well as a chloroplast signal sequence. Phycobilisome binding domain, present in some cyanobacteria was absent. Transcriptome analysis of* Gch* revealed the presence of two Fd; FdL and FdS , sharing the motif CX5CX2CX29X. The analysis indicated that the most probable partner for FNR is FdS.

**Conclusion:**

The interaction model produced, was consistent with functional properties reported for FNR in plants leaves, and opens the possibilities for research in other rhodophyta of commercial interest.

**Electronic supplementary material:**

The online version of this article (10.1186/s40659-017-0144-5) contains supplementary material, which is available to authorized users.

## Background

Ferredoxin NADP (H) oxidoreductases (EC 1.18.1.2) (FNR) are enzymes of 34–45 kDa, involved in crucial steps of photosynthesis in plants, algae and cyanobacteria. Their main function is to provide reduced donors to redox reactions involved in processes such as the fixation of CO_2_ and N_2_, isoprenoids biosynthesis or oxidative stress [1], besides its function in the regulation of the cyclic electron transport in plants [2]. FNR is present in photosynthetic organisms as tissue specific isoforms [3], they co-purify with membrane complexes such as b6f [4], NADPH dehydrogenase [5], Tic62 [6, 7] and Fd in the stroma of chloroplasts [8]. In spinach, FNR is a monomeric enzyme, while in Anabaena, the crystal structure of the complex with Fd shows 2FNR associated to one Fd [9]. In order to perform its function, FNR needs to accommodate the co-factor FAD [10] and its substrates NADP^+^ and ferredoxin. A general characteristic of FNR enzymes, is an optimum pH of 7.0 for the reduction of cytochrome c dependent of ferredoxin at 40–55 °C; under these conditions, for NADPH, K_*m*_ is in the μM order with a turnover number or *k*_*cat*_ of 80–100 s^−1^ [11].

Two domains have been described as a signature of FNRs: a FAD binding domain that includes the N-terminal domain, and a NADP^+^ binding domain that involves the C-terminal domain [12, 13]. In cyanobacteria FNR has been found associated to phycobilisomes (PBS) [14], which is an accessory light harvesting protein complex present in thylakoid membranes. Two isoforms have been described for *Synechocystis* sp.: a small isoform of 33,000 Da, similar to plants stromal FNR, and a large isoform of 45,000 Da, which contains an extra domain at the N-terminal region. The sequence of the extra domain closely resembles a 10 kDa linker protein associated to allophycocyanin in the core of PBS. It has been reported that PBS-associated FNR from *Synechococcus* presents the extra domain [15–18]. Our group has been studying the structure and function of phycobilisomes from *G. Ch*, a red algae that has been commercially cultivated in Chile for agar production. Purified phycobilisomes presented FNR activity. No molecular and functional information is available for FNR from this specie, nor if the protein presents the previously described extra domain. Considering the importance of FNR for mass production of this commercially important algae for polysaccharides and pigments production, this research was focused on the characterization of the enzyme.

Fd and NADP^+^ are the substrates of FNR. Fd is a 11 kDa protein that contains a [2S–2Fe] redox center; in chloroplasts it receives one electron from PSI and transfers it to different enzymes, among them FNR. Considering the sequences, and the type of redox center, low potential (− 420 mV) Fd is present in plants (2Fe–2S) as 90–130 residue proteins, and in bacteria, (4Fe–4S) as 55–100 residue proteins [19]. The electron transfer from Fd to NADP^+^ requires a ternary complex among oxidized FNR, NADP^+^ and reduced Fd, stabilized by hydrophobic interactions and hydrogen bonds, in which the [Fe–S] center of Fd interacts with basic residues in FNR. In Anabaena, Fd interacts with FNR through L76, L78, and V136 at the interface generated by the NADP^+^ binding site and the FAD binding domain [20, 21].

Little information is available regarding the complex FNR/Fd of red algae and especially regarding the eukaryote red algae *G. ch* [22]. Previous results indicated that FNR from *G. ch* is detected in soluble extracts (FNR^SOL^), as well as in purified PBS (FNR^PBS^) [10, 23]. This information leads us to investigate if this FNR could also have the extra domain that enables the binding to PBS, as it is in *Synechococcus.* We report here the sequence of one gene found in the genome of *G. ch*, the sequence analysis of the translated amino acid sequence and the molecular and kinetic characterization of the enzyme. In order to complete the molecular characterization of ferredoxin NADP^+^ reductase from *G. ch*, we also report the sequence of the ferredoxins found in the transcriptome of *G. ch* and molecular models for FNR, its Fd partner and the corresponding FNR/Fd complex.

## Methods

### Purification of phycobilisomes and detection of FNR

Phycobilisomes were purified from 250 g of fresh *G. ch* (Rhodophyta, Gigartinalis)[22] collected in Colcura, Chile (37°6′39″S, 73°8′52″W) according to literature [24, 25] and Additional file 1. The PBS highly enriched fraction was analyzed by non-denaturant 10% polyacrylamide gel electrophoresis [26]. A zymogram was performed to detect FNR diaphorase activity [27]. The procedure involves the incubation of the gel in 50 mM Tris·HCl pH 8, 1 mM EDTA, 0.5 mg mL^−1^ nitro-blue tetrazolium (NBT) as an electron acceptor and 0.5 mM NADPH as substrate donor. The active bands were identified by a blue color appearance after 30 min incubation at 37 °C in the dark. Blue bands were separated and incubated in a denaturant solution and their molecular weights were determined by SDS-PAGE. To detect FNR, Western blots were performed, using anti FNR specific antibodies (Rabbit polyclonal antibodies anti FNR from of *Artrosphira maxima*, 1:1000), generously provided by Dr. Carlos Gómez Lojero, (CINVSTAT, Mexico); a donkey anti rabbit IgG coupled to horseradish peroxidase was used as a second antibody (1:5000) (Jackson ImmunoResearch Laboratories). The peroxidase activity was determined by bioluminescence using PIERCE ECL Western blotting substrate kit. The purification of FNR from the soluble extract (FNR^SOL^) was performed as reported previously [10, 28].

### Determination of kinetic constants

A modified protocol for detecting FNR activity was used [28]. The assay follows the decay of the absorbance at 340 nm due to the oxidation of NADPH in presence of 2, 6-dichlorophenol-indophenol (DCPIP) (Merck). The reaction mixture contained 50 mM Tris·HCl pH 8, 0.15 mM DCPIP, and 1 mM Na_2_EDTA. Variable NADPH concentrations were added to begin the reaction. All the measurements were performed in a Jasco V-650 Spectrophotometer. The activity expressed in μmol min^−1^, was calculated using NADPH ε = 6.220 M^−1^cm^−1^. Initial velocity determinations were performed in triplicate. Kinetic parameters were obtained by fitting the experimental data to the appropriate Michaelis–Menten equation by using nonlinear regression with Graph Pad Prism version 5.0 for Windows (Graph Pad Software Inc., San Diego). *k*_*cat*_ was determined using the protein concentration of FNR in the enriched fraction calculated by densitometric analysis of the SDS-PAGE stained with colloidal Coomassie blue [29].

### Determination of the sequence and cloning of FNR from *Gracilaria chilensis*

Total RNA was obtained using the RNeasy Plant Mini kit (Qiagen, Catalog number 74903). DNAc was obtained by reverse transcription with the M-MLB RT kit (PROMEGA). The amplification of the coding sequence of FNR was performed by Touchdown PCR [30]; specific information is available in Additional file 3. To increase the amount of product, Booster PCR was used [31] and the PCR product of 950 bp was purified using the Zymoclean Gel DNA Recovery Kit (Zymo Research) and cloned into pCR-BluntII TOPO vector (Invitrogen, Life Technologies) by electroporation. Positive clones were selected by kanamycin resistance and cloning was confirmed by enzymatic digestion and sequencing. The 5′UTR sequence was amplified by 5′-RACE using the SMARTer RACE cDNA kit (Clontech), according to the manufacturer. The PCR products were purified and clones were verified as previously described.

### Confirmation of the FNR sequence and search for the sequence of ferredoxin using the transcriptome of *G. ch*

To confirm the sequence of FNR and to obtain the sequence of its partner Fd, we used local Blast against the results of the assembly provided by Trinity [32], for the transcriptome of *G. ch* (AN: SRX1507975) [33]. Two sequences identified as Fd were obtained and used for sequence analysis and structural modeling of the complex. The purified protein was analyzed by MALDI-TOF at the University of Edinburgh and the molecular masses for the tryptic peptides were compared with the translated sequence.

### Sequence analysis and construction of a structural model for FNR

The sequence of FNR was translated in silico to the amino acid sequence using *Translate* (https://web.expasy.org/translate) and it was analyzed using Blastp, tBlastn (https://blast.ncbi.nlm.nih.gov) [34], Pfam (http://pfam.sanger.ac.uk) and ClustalW (http://www.clustal.org) against databases. Subcellular localization and the presence of a chloroplast transit peptide were predicted with ChloroP [35]. A Bayesian phylogenetic reconstruction was performed with MrBayes v3.2.2 and FNR protein sequences from cyanobacteria, plants (both root and leaf isoforms) and red algae, including *G. ch*. Two simultaneous independent runs were conducted using the Jones substitution model, with six parallel chains (one ‘cold’ and five ‘heated’) for 500,000 generations. After a 25% burn-in step, a 50% majority rule consensus tree was calculated with the remaining trees.

The structural model of FNR was obtained with Modeller v9.13 [36] using FNR from *Anabaena*. (PDB code: 1GJR) [37], *Zea mays* (PDB code: 3VO2) [38], *Spinacea oleracea* (PDB code: 1FNB) [39] and *Pisum sativum* (PDB code: 1QFY) [40] as templates. The final step included an energy minimization to eliminate side chain steric clashes by changing the energetically incorrect conformation of several amino acids and improving the hydrogen bond network. The model was evaluated with PROSA [41] and PROCHECK [42] for energetic and stereochemistry assessment, respectively. The model included FAD as a co-factor and NADPH as a ligand and two cycles of Molecular Dynamics. Molecular models for Fd were also produced using the methodology described above for FNR using 2Fe2S Fd from *Mastigocladus laminosus* (PDB code: 1RFK [43] as template, because its sequence identity (64%) and its resolution (1.25 Å). Docking models of Fd with FNR were built with CLUSPRO [44] without restrictions and their interaction surfaces were analyzed with PISA [45].

## Results

**Fig. 1:**
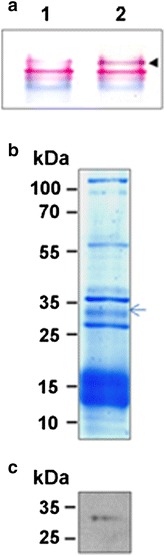
**a** Native PAGE of the purified phycobilisome, lane 1: phycobilisome enriched fraction, lane 2: phycobilisome enriched fraction after diaphorase assay in the gel, **b** SDS PAGE of the PBS enriched fraction, FNR is indicated by an arrow. **c** Western blot for detection of FNR

Purified PBSs were characterized spectroscopically (Additional file 2). The presence of FNR associated to PBSs is shown by its activity in native gels (Fig. 1a) and the Western blot (Fig. 1c). The SDS-PAGE of the PBSs fraction is shown on Fig. 1b.

### Kinetic characterization

K_*m*_^NADPH^ and *k*_*cat*_ were determined for FNR^PBS^ using NADPH as donor substrate and DCPIP as acceptor [46]. The kinetic constants for FNR are shown on Table 1. (Additional file 4) The table also shows the data determined for FNR purified from soluble extract (FNR^SOL^) [10].

**Table 1:** Kinetic constants for FNR in PBSs and in the soluble extract of *G. ch*

Constants	FNR^PBS^	FNR^SOL^
K_*m*_ (μM)	12.5 ± 1.8	16.3 ± 0.3
k_cat_ (s^−1^)^a^	86	56.1
Catalytic efficiency (μM s^−1^)	6.9	3.4

The same procedure was used to determine the kinetic constants for the semi purified enzyme from the soluble extract and for the enzyme that co-purified with phycobilisomes

^a^Protein concentration was determined by densitometry in SDS polyacrylamide gels using bovine serum albumin (BSA) (Sigma-Aldrich) as standard

### The sequence

**Fig. 2:**
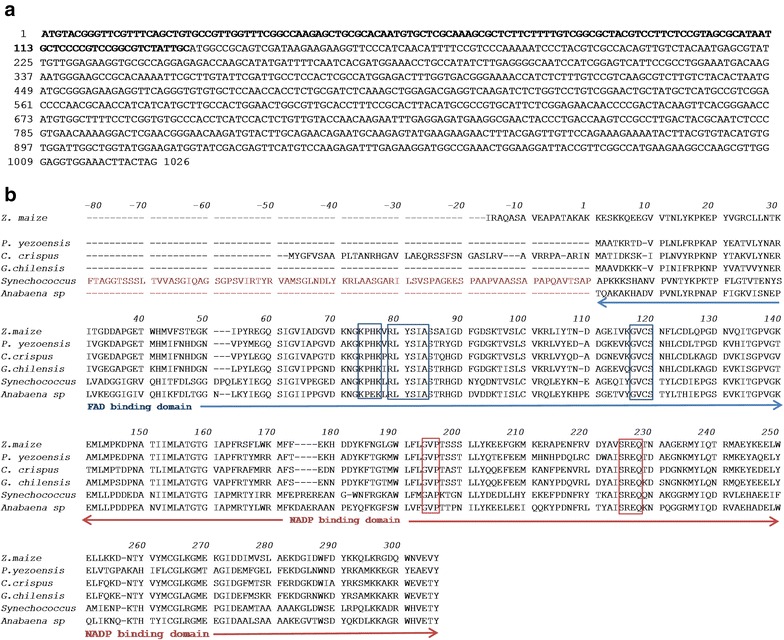
**a** Nucleotide sequence of the gene identified for FNR of *Gracilaria chilensis*. Nucleotides 1–135 (bold letters) codify for a chloroplastide transit signal. **b** Translated sequence of the *G. ch* mature protein, from amino acid 1. A sequence alignment with FNR from *Zea mays, Pyropia yezoensis, Chondrus crispus, Synechococcus* sp. and *Anabaena* sp.* is also shown. In *Synechococcus*, the sequence for a PBS binding third domain is shown (residues – 1 to − 80). The NADP and FAD binding domains are indicated by arrows, the conserved residues for the binding of co-factor and substrate are also shown in blue squares and red squares for FAD and NADP binding residues respectively.Amino acid residues involved in the interaction with ferredoxin are also shown. *The third domain for *Anabaena* sp. is not shown for the clarity of the alignment. Only the common two domains present in the structural complex (1ewy) are shown

The sequencing experiments, including the elongation of the 5′ coding region by 5′RACE, provided the expected product of 1026 bp, corresponding to the sequence shown on Fig. 2a which includes a chloroplast coding segment (nucleotides 1–135). The 5′RACE results as well as the sequence provided from the *G. ch* transcriptome analysis indicate the absence of CpcD domain. The calculated pI for the FNR from *G. ch* was 6.26 and the calculated molecular mass was 33,646.16 Da. The absence of the Cpc Domain was confirmed by mass spectrometry, in which the mass of a peptide corresponding to the N terminal sequence MAAVDKKK (1–8) was detected as well as the peptide VPINIFRPK (9–17). The analysis confirmed the 100% of the residues reported for the mature protein. The Cpc domain was not detected.

### Sequence analysis and structural homology model of FNR

**Fig. 3:**
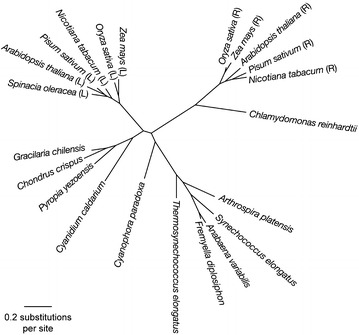
Unrooted phylogenetic tree built using the sequences of *Gracilaria chilensis* (Rhodophyta, eukaryote) *Chondrus crispus* (Rhodophyta, eukaryote), *Pyropia yezoensis* (Rhodophyta, eukaryote), *Cyanidium caldarium* (Rhodophyta, eukaryote), *Cyanophora paradoxa* (Glaucophyta, eukaryote), *Thermosynechococcus elongates* (Cyanobacteria, prokaryote), *Fremyella diplosiphon* (Cyanobacteria, prokaryote), *Anabaena variabilis* (Cyanobacteria, prokaryote), *Synechococcus elongates* (Cyanobacteria, prokaryote), *Arthrospira platensis* (Cyanobacteria, prokaryote), *Chlamydomonas reinhardtii* (Chlorophyta, eukaryote), *Nicotiana tabacum* (Magnoliophyta, eukaryote), *Pisum sativum* (Anthophyta, eukaryote), *Arabidopsis thaliana* (Tracheophyta, eukaryote), *Zea mays* (Magnoliophyta, eukaryote), *Oryza sativa* (Magnoliophyta, eukaryote), and *Spinacia oleracea* (Streptophyta, eukaryote). Letters in parenthesis indicate leaf (L) or root (R) isoforms

**Fig. 4:**
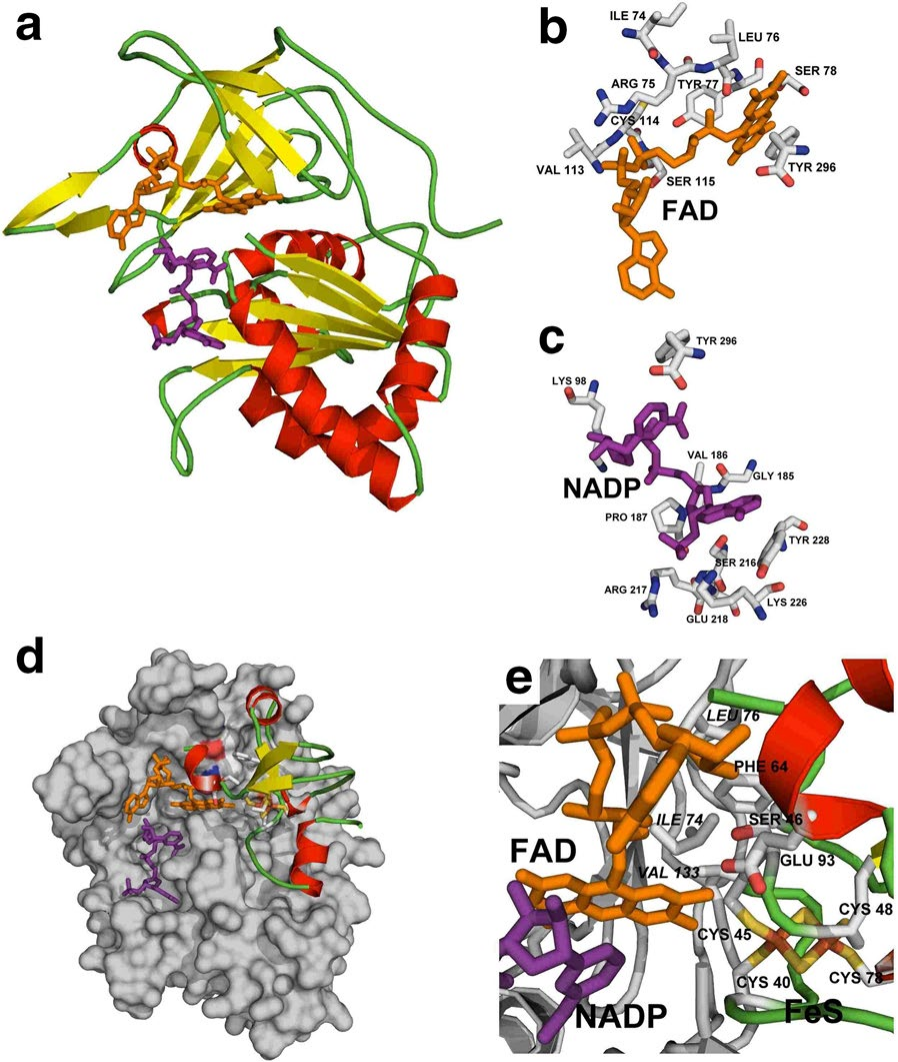
**a** Comparative model of ferredoxin NADP^+^ reductase from *Gracilaria chilensis.* Secondary structures are shown by arrows (β strands) and cylinders (α helices); FAD is shown as orange sticks and NADP as purple sticks. **b** Close up of the residues involved in the NADP^+^ binding site, **c** close up of the residues involved in the FAD binding site. **d** Docking model of FNR (grey surface), showing the two co-substrates, NADP^+^ and ferredoxin, the co-factor FAD and the 2[FeS] cluster. **e** Close-up of the complex showing residues of the interface included in the text

The sequence of 298 amino acids for the mature protein of *G. ch* in a multiple alignment with FNR from the plant *Z. mays* (2 domains enzyme), from the cyanobacteria *Synechococcus* sp. *(*three domains enzyme) and *Anabaena* (three domains enzyme, the third domain is not shown in the alignment), from the red algae *Pyropia yezoensis* and *Chondrus crispus,* is presented on Fig. 2b. Both cyanobacterial FNR present an N-terminal CpcD domain, which is absent in *Z. mays* and red algae FNRs. The alignment analysis on Fig. 2b shows the presence of the FAD and NADP^+^ binding domains, a signature for the FNR family. The residues indicated as belonging to NADP^+^ and FAD binding domains in the alignment are also conserved in most of the sequences of FNR; the corresponding motifs in *G. ch* are: 71–84 (RLYSIA) and 117–121 (GVCS) for the FAD binding region and 194–196 (GVP) and 225–227 (SRE) for the binding of NADP^+^. To be able to capture electrons from Fd, FNR forms a ternary complex that includes NADP^+^. In this complex some residues, such as (KPHK) in *Synechococcus* sp. (residues 74–77 in *G. ch* FNR), have been also reported to interact with Fd [47]. The phylogenetic analysis produced the tree shown in Fig. 3, in which 4 monophyletic clades of FNR sequences are clearly detected: cyanobacteria, plant leaves, plant roots, and red algae, in which the *G. ch* sequence of FNR is included. To build a model for the enzyme of *G. ch*, Modeller v.9.13 was used with the templates mentioned previously. The model proposed for FNR, shown on Fig. 4a, was stereochemistry and energetically stable. It shows two domains that have been described for plants FNR a: the FAD binding domain, formed by six antiparallel β strands organized in a β-barrel with a greek-key topology, that provides the backbone for the binding of the FAD molecule with the isoalloxazine ring located between the two domains, and b: the NADP^+^ binding domain, formed by three-layer sandwich α/β/α with a Rossmann-like topology and a parallel five membered β sheet stabilized by six helices [48]. As it was reported for other FNRs, C-terminal Y (306 in the alignment shown in Fig. 2b in *G. ch*), is part of the binding site and it has been proposed that it occupies the nicotinamide catalytic binding site in the free enzyme [49, 50]. The binding sites are presented in Fig. 4b, c as observed in the molecular model. The highlighted residues in the alignment (Fig. 2b) are labeled in Fig. 4b, c. Most of the residues forming the FAD and NADP^+^ binding site are conserved and occupy similar positions in the FNR structures.

### The co-substrate, ferredoxin

**Fig. 5:**
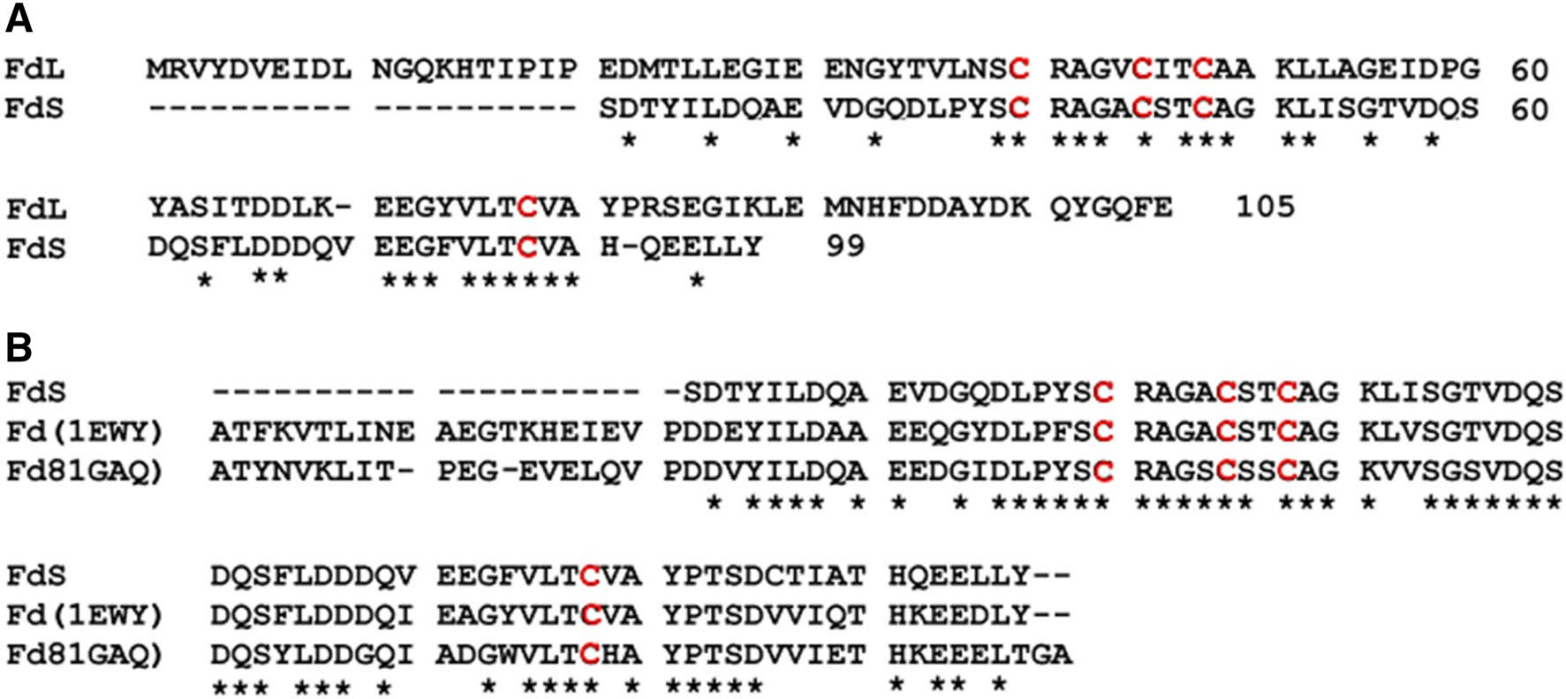
Sequences of ferredoxins as detected in the transcriptome of *Gracilaria chilensis.* **a** Alignment of the FdS with FdL. **b** Sequence alignment among FdS and ferredoxins from *Z. mays* (1gaq) and *Anabaena* sp. (1ewy). Only the identities are shown as (*), the cysteines involved in the binding of the FeS center are displayed in red

Information from transcriptome assembly produce two sequences identified as Fd (FdL = large ferredoxin, and FdS = short ferredoxin); both shared the motif CX5CX2CX29C, corresponding to plant Fds [51] and they are 41% identical. The translated sequences are shown on Fig. 5. The sequence for FdS was not found complete in the transcriptome but from its high identity with *Z. mays* Fd (79%) and *Anabaena* Fds (71%), it is reasonable to propose it corresponds to a short plant ferredoxin of 99 amino acids. FdL also belongs to the family of plants ferredoxins and has 105 amino acid residues.

### Structural homology model for FdS, and the complex FNR/FdS

In order to review the interaction surfaces in the protein complex, our analysis also included the study of the selected FNR partner FdS, which shares 79 and 71% identity with Fds present in the complexes FNR/Fd reported at the Protein Data Bank from *Z. Mays* (PDB code: 1GAQ) [38] and *Anabaena* (PDB code: 1EWY) [37], respectively (Fig. 5b). FdL shares only 29 and 36% identity with Fd from the sequences in the complexes reported for *Z. Maize* and *Anabaena* respectively. Thus, FdS was chosen as an adequate partner for FNR.

A molecular model for FdS is shown Fig. 4c as a partner for FNR. The model for FdS presents three helical regions (H1: I25–D32; H2: D67–E71; H3: E93–Y96) that flank the interaction surface and three beta strands (SA: A48–S54; SB: F74–L76, SC: T87–A89), which seems to contribute to the hydrophobic steadiness around the 2F–2S cluster. The cysteines that maintain the cluster in the correct position are C40, C45, C48 and C78, following the numbering in the structural alignment showed in Fig. 5b. The secondary structure described in FdS supports a less structured region facing the interface with FNR. The essential amino acid residues for the activity have been reported as S46, F 64 and E93 in *Anabaena* Fd [38], these residues are also present in equivalent positions in *G. ch* FdS, as well as in *Z. mays,* in which F64 is replaced by Y64 [37] performing an identical function.

The high ranked interaction model obtained with Cluspro [44] for the partners FNR and FdS is shown on Fig. 4c, d. The model was analyzed with PISA [44] revealing that 10.5% of FNR residues and 37.3% of FdS residues are involved in the interaction surface corresponding to 865 and 980 Å^2^ respectively. The interaction shows that electrostatic interactions are important for the stabilization of the complex as they are in the complexes reported for *Zea mays* and *Anabaena* (PDB codes: 1GAQ, 1EWY) [37, 38]. The interacting model shows that F64 in the hydrophobic core is close to the FeS cluster and to the isoalloxazine ring of the cofactor FAD. It has been described that an aromatic residue in that position is important for the stability of the hydrophobic core, which is also formed by I74, L76, and V133 in FNR. The distance between the C8 methyl of FAD and the FeS cluster is 7.4 Å which corresponds fairly well with the distances found in 1GAQ and 1 EWY. This distance is consistent with the evidence that suggest that C8 is involved in the energy transfer.

## Discussion

Algae and cyanobacteria depend on their light harvesting systems to survive. Phycobilisomes are the principal auxiliary light harvesting protein complexes in these organisms. The analysis of PBS had revealed previously the presence of FNR in the proximity of PBS and PSI in *Synechococcus* [15]. FNR has been extensively studied in plants, but not so frequently in eukaryotic alga. In plants and cyanobacteria, FNR contains at least two domains: the FAD binding domain and the NADP binding domain [28]. In cyanobacteria, an additional domain has been described, whose sequence is homologous to a PBS linker (CpcD), suggesting a role of PBS binding domain. In the red alga *Gracilaria chilensis*, we expected to find a similar domain considering that FNR activity was detected in purified phycobilisomes, as well as in soluble extracts (FNR^SOL^) [10]. However, the detected FNR had a molecular weight of 34,000 Da, which accounts only for the FAD and NADP^+^ binding domains. The phylogenetic tree shown on Fig. 3 indicates a closer proximity of FNR from *G. ch* with FNR from leaves in plants (FNRL) than with those from cyanobacteria. This could be related to absence of the third domain.

The kinetic characterization performed with FNR^PBS^ present in PBS enriched fractions, at high salt concentration in order to avoid PBS dissociation, showed also similarity with two domains FNR from plants. The K_*m*_^PBS^ for NADPH was 12.5 μM, similar to the K_*m*_^Sol^ = 16.3 μM obtained for the FNR purified previously from soluble extract in our laboratory. In addition, the k_*cat*_ values in both samples are similar. These values agree with K_*m*_ reported for two domains FNR in general and with leaves FNR [52, 53]. It has been also reported that the association of the CpcD domain in *Synechocystis* FNR with Phycocyanin, does not change the catalytic efficiency [54]. Nevertheless, in that study as well as in ours, no other components of the phycobilisome were considered [55]. The molecular weight (MW) suggests the absence of the CpcD domain.

To address the possibility that a 3 domain protein could exist temporarily, we looked for the nucleotide sequence of the gen in total DNA. To date, only one gene that codifies for FNR (petH) has been identified in eukaryotes and cyanobacteria and it has been proposed that MW variants are a product of proteolytic cleavage of cpcD domain or different reading frames [56]. To design the primers to clone the gen in the *G. ch* genome, a bioinformatic study was performed using the chloroplast DNA information corresponding to an EST library for *Gracilaria tenuistipitata* [57]. This sequence did not contain the cpcD domain as reported for *Porphyra yezoensis* by other authors [58]. Actually, the *G. ch* FNR amino acid translated sequence showed more similarity with FNR present in plants leaves than FNR from roots and Cyanobacteria, as shown by a Bayesian analysis (Fig. 3). It has been proposed that the presence of the CpcD domain of FNR associated to PBS in cyanobacteria would help the location of the enzyme in the vicinity of PSI to capture electrons from Fd, nevertheless if this function occurs also in *G. ch* FNR, the mechanism for the association of FNR to PBS should be different.

As described in results, the transcriptome analysis detected two Fd sequences, that we called FdS and FdL. Both belong to plants Fds type [51]. FdS showed highest identity with the two ferredoxins in the complexes FNR/Fd reported in the protein data bank, so we use FdS as co-substrate for FNR. Considering all the previous information, and using the sequence just reported, the optimized model of FNR satisfied all the energetic and stereo-chemical requirements, and it accommodated well the FAD and NADP^+^ binding sites; the amino acid residues that are important for the function were all present in the model. This model and the model obtained for FdS from *G. ch* were used to build an interaction model whose architecture is, in general, very similar to the three dimensional structures reported for the complexes: the two domains FNR/Fd for *Z. mays* and *Anabaena* (without considering the PBS binding domain). The important residues for the activity, for the binding of the co-factor FAD and the substrates NADP and ferredoxin, are all present and in the correct geometry to perform the function.

In summary, FNR from *Gracilaria chilensis* shows high similarity with two domains enzymes from plants and red alga. There is a possibility that FNR could be associated to phycobilisomes, which has been described for *Synechocystis* (three domains enzyme) [54]. On the other hand, the possibility of interaction of FNR with the chloroplastidial membrane anchor proteins Tic62 [59] or Trol, a rhodanase like protein, responsible for the docking of FNR [60], as it has been found in *Arabidopsis,* should not be discarded; recent data show that Trol is necessary to the dynamic recruitment of FNR to membranes [61].

This and other possibilities can account for the reductase activity detected for FNR^SOL^ and FNR^PBS^. Phycobilisomes function is to harvest and transfer energy towards photosystems, function that also generate redox species that also need to be eliminated for protection of the light dependent processes, so it is possible that besides its binding to FdS, FNR could be associated to different partners such flavodoxins or other oxidoreductases.

## Conclusions

The nucleotide sequence for one FNR gene from *G. ch*, was sequenced and translated to a protein of 33,646 Da. Sequence analysis identified a FAD and a NADP^+^ binding domain, as well as a chloroplast signal sequence. FNR from *G. ch* lacked the PBS binding domain, which is present in some cyanobacteria. Transcriptome analysis of *G. ch* revealed the presence of two Fds; FdL (large) and FdS (short), sharing the motif CX5CX2CX29X. The sequences and the structural analysis reported here, indicate that the most probable partner for FNR in *G. ch* is FdS. The interaction model produced is consistent with functional properties reported for FNR in plants leaves.

## Additional files

**Additional file 1.** PBS purification protocol.

**Additional file 2.** Spectroscopic characterization of purified phycobilisomes.

**Additional file 3.** Specific information regarding the determination of the sequence and cloning of FNR from *Gracilaria chilensis*.

**Additional file 4.** Saturation curve and Lineweaver-Burk plot for the enzymatic activity of FNR present in PBS of* Gracilaria chilensis*.
