# Supplementary material for: Molecular and functional characterization of ferredoxin NADP(H) oxidoreductase from Gracilaria chilensis and its complex with ferredoxin
Source: Biol Res. 2017 Dec 8;50:39. doi: 10.1186/s40659-017-0144-5 (PMC5723097; doi:10.1186/s40659-017-0144-5)
Supplement: Supplementary file 1 — Additional file 1. PBS purification protocol. [file 40659_2017_144_MOESM1_ESM.docx]

Additional file 1

PBS purification protocol

250g of fresh *Gracilaria chilensis* sp C.J.Bird, McLachlan & E.C.Oliveira (Rhodophyta, Gigartinalis) collected in Colcura, Chile (37^o^6’39’’S, 73^o^8’52’’W) was frozen with N_2_(l), powdered in a mortar and suspended in lysis buffer: 1M Phosphate buffer pH 7.5 containing 2%v/v Triton X-100 and 0.05%v/v protease inhibitors (Protease Inhibitor Cocktail P9599 SIGMA-ALDRICH, 1mM PMSF,1mM EDTA). The mixture was incubated for 2h at room temperature in the dark. The soluble material was separated by filtration on cheesecloth and centrifuged at 27000 x g for 30min at 4ºC. The supernatant was ultracentrifuged at 117504 x g for 3h at 4ºC. The pellet was suspended in a minimum amount of sample buffer (0.9M Phosphate buffer pH 7, 2% v/v Tritón X-100, 0.05%v/v with Protease Inhibitor Cocktail) and it was stirred overnight at 4ºC and darkness. A phycobilisome rich fraction was obtained by a discontinuous sucrose gradient (0.5, 0.8, 1.2, and 2M) centrifuged at 162635 x g for 5h at 4ºC. The colored fractions were separated and characterized by spectroscopy to determine their composition (Bruna, 2001).
