# Supplementary material for: Molecular and functional characterization of ferredoxin NADP(H) oxidoreductase from Gracilaria chilensis and its complex with ferredoxin
Source: Biol Res. 2017 Dec 8;50:39. doi: 10.1186/s40659-017-0144-5 (PMC5723097; doi:10.1186/s40659-017-0144-5)

*Additional file 2*

*Spectroscopic characterization of purified phycobilisomes*

The figure below shows A) the absorption spectrum of a purified phycobilisome showing the maxima corresponding to the phycobiliproteins: phycoerythrin, phycocyanin and allophycocyanin. In B) the fluorescence intensity (FI) spectra for the phycobilisome excited at 566, 620 and 650nm is shown. The emission corresponds to 661nm which is a test for complete phycobilisomes.


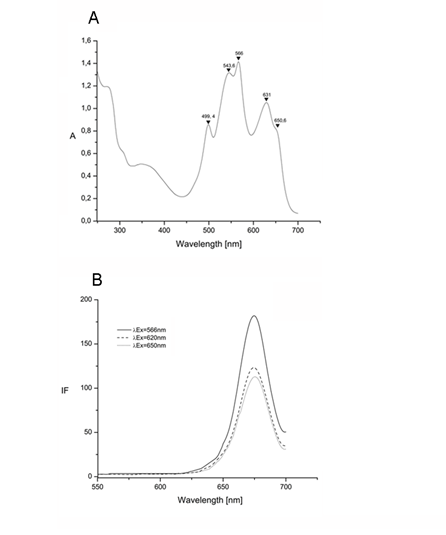

Supplement: Supplementary file 2 — Additional file 2. Spectroscopic characterization of purified phycobilisomes. [file 40659_2017_144_MOESM2_ESM.docx]
