# Supplementary material for: Molecular and functional characterization of ferredoxin NADP(H) oxidoreductase from Gracilaria chilensis and its complex with ferredoxin
Source: Biol Res. 2017 Dec 8;50:39. doi: 10.1186/s40659-017-0144-5 (PMC5723097; doi:10.1186/s40659-017-0144-5)
Supplement: Supplementary file 3 — Additional file 3. Specific information regarding the determination of the sequence and cloning of FNR from Gracilaria chilensis. [file 40659_2017_144_MOESM3_ESM.docx]

*Additional file 3*

*Specific information regarding the determination of the sequence and cloning of FNR from Gracilaria chilensis*

*Primers:* Reverse primer 5’CGAGATATGTCGAATAAGATGTC-3’ (corresponding to the 3’UTR from *G.Tenuistipitata)* was used for reverse transcription and touchdown PCR. Primer sense 5’ATGGCCGCAGTCGATAAG-3’ for touchdown PCR. FNR specific reverse primer 5'-TCCATGGCGAGTGGAGGCAATCGAATAC-3' was used for 5'-RACE. Internal FNR-specific primer 5'-TCCGATGGATTGCCCCTCAAGAT-3' was used for nested-PCR.

*Touchdown PCR for sequencing and cloning FNR*: M.J.Research PTC 100 thermocycler, TAKARA ExTaq enzyme, 2μg of template and 10μM of sense and antisense primers were used.

*5'-RACE for determining the 5’UTR of FNR*: 500 ng of total RNA purified using Direct-Zol RNA extraction kit (Zymo Research) were used for cDNA synthesis, and touch down PCR amplification (ExTaq Hot Start DNA polimerase, TAKARA) was performed using the FNR specific reverse and the universal primer mix provided with the kit. The PCR product was employed as template in a nested-PCR amplification reaction.
