# Supplementary material for: Molecular and functional characterization of ferredoxin NADP(H) oxidoreductase from Gracilaria chilensis and its complex with ferredoxin
Source: Biol Res. 2017 Dec 8;50:39. doi: 10.1186/s40659-017-0144-5 (PMC5723097; doi:10.1186/s40659-017-0144-5)
Supplement: Supplementary file 4 — Additional file 4. Saturation curve and Lineweaver-Burk plot for the enzymatic activity of FNR present in PBS of Gracilaria chilensis. [file 40659_2017_144_MOESM4_ESM.docx]

Additional file 4

Saturation curve and Lineweaver-Burk plot for the enzymatic activity of FNR present in PBS of *Gracilaria chilensis*. A) Dependance of the initial rate (Vi), on the NADPH concentrations ( 5, 7, 12.5, 20, 80, 100, and 120μM) at a constant concentration of DCPIP( 0.15mM). B) Double reciprocal plot of the data in A.
